# Cytotoxic Sesquiterpene Lactones from *Kauna lasiophthalma* Griseb

**DOI:** 10.3797/scipharm.1310-18

**Published:** 2014-01-18

**Authors:** Eliana M. Maldonado, Daniel Svensson, Stina M. Oredsson, Olov Sterner

**Affiliations:** 1 Centre for Analysis and Synthesis, Lund University, Getingevägen 60, 221 00, Lund, Sweden.; 2 Centro de Tecnología Agroindustrial, San Simón University, Cochabamba, Bolivia.; 3 Departament of Biology, Lund University, Sölvegatan 35A, 223 62, Lund, Sweden.

**Keywords:** *Kaunia lasiophthalma*, Cytotoxicity, Sesquiterpene lactones, Breast cancer cell lines

## Abstract

Two new eudesmane derivatives (**3** and **8**) were isolated from the ethanol extract of the aerial parts of *Kaunia lasiophthalma* Griseb, together with 14 known eudesmane, germacrane, and guaiane sesquiterpenes, and four flavones. The structures and relative configurations of all the compounds were established by NMR spectroscopy and high-resolution mass spectrometry. The anticancer activity of sesquiterpenes **1**, **3**, **6**–**9**, **11**, **12**, **14**, and **16** was evaluated *in vitro* with the breast cancer cell lines HCC1937, JIMT-1, L56Br-C1, MCF-7, and SK-BR-3, and compared with the cytotoxicity in the non-cancerous breast epithelial cell line MCF-10A. All compounds were found to possess anticancer activity, and compound **1** was the most potent in all of the investigated cancer cell lines with IC_50_ values ranging between 2.0 and 6.2 μM. In order to demonstrate the importance of the α-methylene-γ-lactone/ester moiety present in all compounds for the effects on the cells, the methyl cysteine adduct **21** was prepared from **9** and found to be inactive or considerably less potent.

## Introduction

The relatively small genus *Kaunia* (Asteraceae: Eupatorieae) comprises only 14 species of which most grow in Bolivia, but that also are found in Argentina, Brazil, Peru, and Ecuador [1, 2]. Previous chemical investigations of plants classified in this genus have revealed the presence of sesquiterpene lactones, predominately guaianes, and thymol derivatives as the main constituents [3–5]. *Kaunia lasiophthalma* Griseb *(syn. Eupatorium lasiophthalmum* G.) is a shrub bearing white-purple flowers, locally known in Cochabamba, Bolivia by the common name of “Tuwi” and used to treat inflammation and headaches (Lic. Modesto Zárate, personal communication). It has previously been subjected to a phytochemical investigation by Gutierrez and co-workers, who isolated 19 guaiane sesquiterpene lactones [6]. In our search for biologically active compounds in the Bolivian flora, we have isolated and characterized the chemical constituents of the aerial parts of *K. lasiophthalma* G. Two new eudesmanolide derivatives, compounds **3** and **8**, together with 18 known compounds (**1**, **2**, **4**–**7, 9**–**20**) were obtained, and this constitutes the first report of eudesmanolide and germacranolide sesquiterpenes as well as flavones from this species. As most isolated compounds possess an α-methylene-γ-lactone moiety that previously has been associated with various biological activities, we decided to investigate the effects of the sesquiterpenes available in sufficient quantities on cancer cells. Unsaturated lactones are likely to exert biological effects because they react with cell constituents as Michael acceptors, and have consequently been considered to be generally toxic. However, recent studies have shown that Michael acceptors may be selective and several are currently in clinical trials as drug candidates [7].

Human breast cancer can be classified into different molecular subtypes using gene expression profiles [8–13]. The anticancer activity of compounds **1**, **3**, **6–9**, **11**, **12**, **14**, and **16** in the five breast cancer cell lines MCF-7 (luminal A subtype), SK-BR-3 (luminal B), HCC1937, L56Br-C1 (basal subtype), and JIMT-1 (HER2 subtype) was compared with the cytotoxicity in the normal-like breast epithelial cell line MCF-10A [8–13]. In breast cancer, each subtype has a different prognosis and is subjected to different treatment. The luminal A and B subgroups express estrogen receptors and are amenable to hormone therapy, while the HER2 group, expressing the human epidermal growth factor receptor 2, may be subjected to trastuzumab therapy. The basal tumors lack expression of both estrogen receptors and HER2; they are biologically more aggressive and the prognosis is often poorer.

## Results and Discussion

**Fig. 1:**
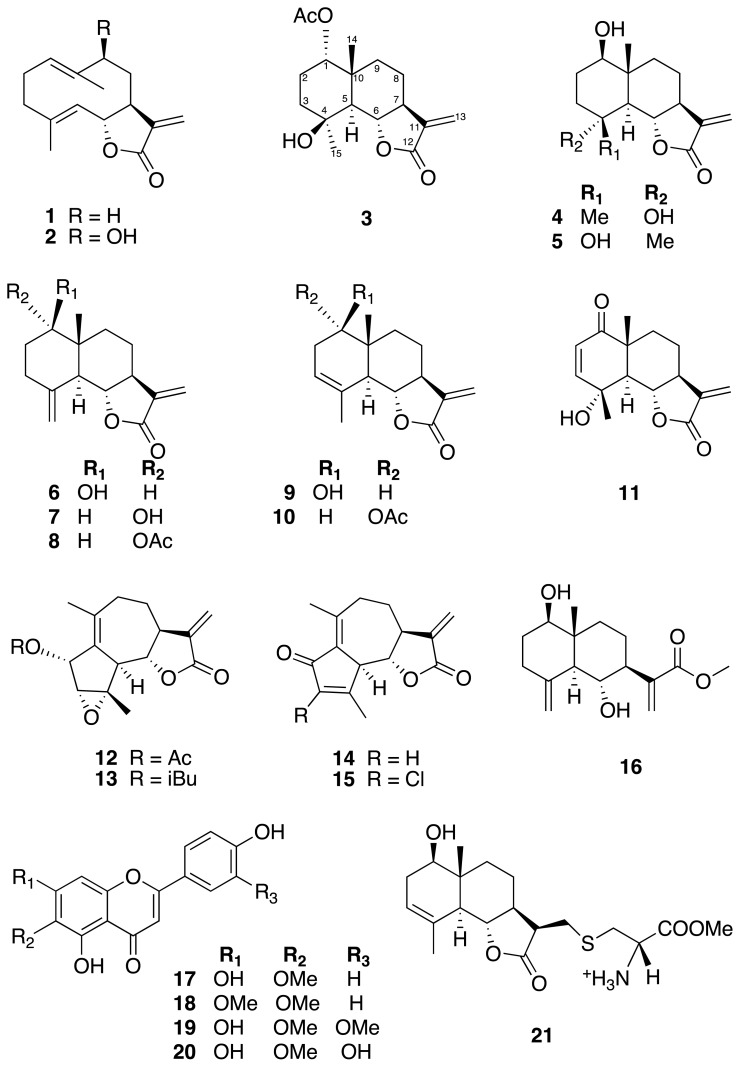
Structures of the isolated compounds from *K. lasiophthalma* **1–20** and methyl cysteine adduct **21**

The EtOH extract of the leaves and flowers of *K. lasiophthalma* G. were subjected, separately, to sequential liquid-liquid partition with hexane, CH_2_Cl_2_, and EtOAc. The major constituents were found to be fats, which were not further investigated, and the compounds reported here are minor metabolites of this plant. Vacuum liquid chromatography (VLC) of the CH_2_Cl_2_ fractions followed by silica gel and Sephadex LH-20 chromatography as well as HPLC fractionation afforded the two new natural products **3** and **8,** along with 18 known compounds, 14 sesquiterpenes and four flavonoids (see Figure 1 for chemical structures).

The known compounds isolated from leaves (**1**, **6**, **7**, **9**, **10**, **16**–**19**) and flowers (**1**, **2**, **4**–**7**, **9**–**16**, **20**) were identified by combined spectroscopic analyses and comparison with literature data, as costunolide (**1**) [14], haageanolide (**2**) [15], 1β-hydroxyarbusculin A (**4**) [16], 4-*epi*-1β-hydroxyarbusculin A (**5**) [17], reynosin (**6**) [18], 1-*epi*-reynosin (**7**) [19], santamarin (**9**) [20], the acetate of santamarin (**10**) [21], 11,13-didehydrovulgarin (**11**) [22], 2β**-**acetoxy-3α,4α-epoxy-3,4-dihydrokauniolide (**12**) [6], 3α,4α-epoxy-2α-isobutyryloxykau-niolide (**13**) [6], dehydroleucodin (**14**) [23], 3-chlorodehydroleucodin (**15**) [6], baynol C (**16**) [24], hispidulin (**17**) [25], cirsimaritin (**18**) [26], jaceosidin (**19**) [27], and eupafolin (**20**) [28].

**Fig. 2:**
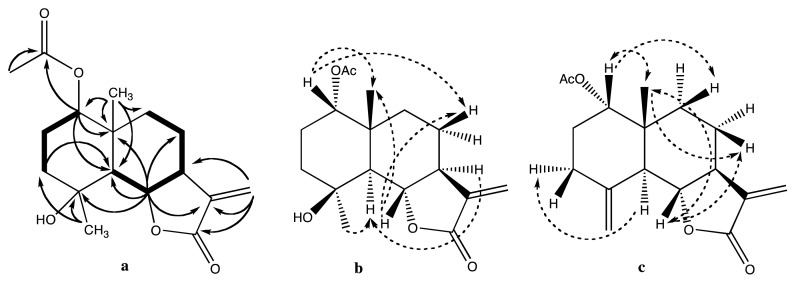
(a) Important HMBC (arrow) and ^1^H-^1^H COSY (bold) correlations for **3** (b) and (c) Key NOESY correlations for **3** and **8**, respectively

Compound **3** was obtained as a colourless gum. The HR-ESI-MS indicated that its elemental composition is C_17_H_25_O_5_, which suggests six degrees of unsaturation. The IR spectrum showed absorption bands corresponding to a hydroxyl group (3473 cm^−1^), an α,β-unsaturated-γ-lactone function (1764 cm^−1^), and an ester group (1730 cm^−1^). The NMR spectra displayed the characteristic signals of an eudesmane lactone, see Table 1 for 1D NMR data and Figure 2 for 2D data. The presence of an exomethylene-γ-lactone ring was established by the 13-H_2_ proton signals at δ_H_ 5.94 and 4.79 and their HMBC correlations to C-7, C-11, and C-12, and the 6-H lactone proton signal at δ_H_ 3.70 and its strong ^1^H-^1^H coupling with 7-H and HMBC correlation to C-12. In addition, the NMR data indicated the presence of the acetoxylated tertiary carbon (C-1) at δ_H_ 4.67 and δ_C_ 77.2 and HMBC correlations from both 1-H and acetoxy-H_3_ to the acetoxy carbonyl carbon (C-1′), and an oxygenated quaternary carbon (C-4) at δ_C_ 71.0. The complete structural elucidation of **3** was achieved by analysis of the HMQC, HMBC, and COSY spectra. The large ^1^H-^1^H coupling constant between 5-H and 6-H and the HMBC correlations between 14-H_3_ and C-1, C-5, C-9, and C-10 links C-5 and the adjacent γ-lactone to the quaternary C-10. COSY correlations from 1-H via 2-H_2_ to 3-H_2_, and HMBC correlations from 1-H to C-5 and C-10, from 6-H to C-4, C-5, C-8, and C-10, as well as from 15-H_3_ to C-3, C-4, and C-5 close the left cyclohexane ring. The ^1^H-^1^H spin system 5-H/6-H/7-H/8-H_2_/9-H_2_ together with HMBC correlations from 6-H to C-8 and from 7-H to C-9 close the second cyclohexane ring and establish the eudesmane skeleton unambiguously. The relative configuration of **3** was suggested by correlations observed in the NOESY spectrum, from 14-H_3_ to 1-H, 2β-H, 3β-H, 6-H, and 8β-H, as well as from 5-H to 2α-H, 3α-H, 7-H, 8α-H, and 15-H_3_. The proposed configuration is confirmed by the large coupling constants between 5-H and 6-H as well as 6-H and 7-H, showing that the three protons are axial, while the small coupling constants between 1-H and 2-H_2_ show that 1-H is equatorial. The comparison of the spectroscopic data of compound **3** with those of 4-*epi*-1β-hydroxyarbusculin (**5**) [17] indicated that they are similar. The difference is that the C-1 hydroxyl group in **5** is acetylated in **3** and that the configuration at C-1 is inversed, which consequently identifies **3** as 4-*epi*-1α-acetoxy arbusculin A.

Compound **8** was isolated as clear oil. The elemental composition was established by HR-ESI-MS to be C_17_H_22_O_4_, indicating seven degrees of unsaturation. The IR spectrum exhibited the presence of an α,β-unsaturated-γ-lactone (1770 cm^−1^) and an ester group (1726 cm^−1^). The ^1^H NMR data of **8** are similar to those of the acetate of reynosin [29], the significant differences are the chemical shift of 1-H and 5-H as well as the coupling constants between 1-H and 2-H_2_ which are small in **8** as 1-H is equatorial, but large for the acetate of reynosin as 1-H is axial. An analysis of the NOESY spectrum of **8** showed correlations from 14-H_3_ to 1-H, 2β-H, 6-H, 8β-H, and 9β-H as well as from 5-H to 3α-H, 7-H, and 8α-H. Therefore, compound **8** was identified as acetyl 1-*epi*-reynosin.

The anticancer activities of sesquiterpenes **1**, **3**, **6**–**9**, **11**, **12**, **14,** and **16** were assessed in five breast-cancer cell lines, HCC1937, JIMT-1, L56Br-C1, MCF-7, and SK-BR-3, and compared with the cytotoxicity in the breast-derived non-cancerous cell line MCF-10A using the MTT colorimetric assay. The inhibitory concentration 50 values (IC_50_) were deduced from the obtained dose-response curves and are presented in Table 2. Interestingly, the cancer cell lines were more sensitive to all of the compounds than the normal-like MCF-10A cells. No obvious patterns related to the breast cancer cell line subgroup (*vide supra*) was found, and all compounds possessed activity. Compound **1** was found to be the most active in all of the cell lines with IC_50_ values ranging from 2.0 to 6.2 μM in the cancer cell lines, while compounds **3** and **16** exhibited the lowest activity. **16** is an unsaturated ester and differs in that respect from the other compounds, but the lower activity of **3** compared to the similar compounds was unexpected. Costunolide (**1**) differs from the eudesmane sesquiterpenes by having the unsaturated lactone fused with a macrocyclic system instead of a cyclohexane ring, and the tension of the lactone ring is likely to be lower in **1**. This would render **1** less reactive and possibly more selective. Indeed, **1** together with the two guaianes **12** and **14**, shows a slightly higher selectivity for the cancer cells compared to the eudesmanes. The difference in the activities of **7** and **8** may depend on the higher lipophilicity of **8**, facilitating its absorption into the cells. In **11** and **14**, the presence of a second Michael acceptor function may influence the activity. It is difficult to speculate from these data, but a trend is that the MCF-7 cells seem slightly less affected than the other cancer cell lines. MCF-7 has a normal wild type *p53* gene, which the MCF-10A cells also have, while the others have a mutated *p53*. Thus, MCF-7 and MCF-10A cells may share a property of being blocked in the G_1_ phase of the cell cycle, which has a protective function, while the other cancer cell lines do not.

Previous investigations of sesquiterpenoid α-methylene-γ-lactones have indicated that the cytotoxic and antitumor activities are related to their ability to react as Michael acceptors [7]. We therefore added methyl cysteine to the exocyclic methylene group of **9** to give **21**, and compared its cytotoxicity with the natural products’. As can be seen from the results in Table 2, **21** is significantly less potent, however, it is not devoid of activity and its cytotoxicity towards the normal-like MCF-10A cells is similar to that of the α-methylene-γ-lactones. This may depend on the reversibility of Michael additions, by which **21** slowly can eliminate methyl cysteine and regenerate **9** during the assay conditions [7].

In conclusion, the molecular causes for the lower cytotoxicity of the compounds (especially compound **1**) in the normal-like breast epithelial MCF-10A compared to the breast cancer cells lines need to be further exploited and may find clinical use by showing less off-target cytotoxicity.

## Experimental

### General

Optical rotations were measured with a Perkin Elmer Model 341 polarimeter. IR spectra were recorded with a Bruker Alpha-P FT-IR instrument in the ATR geometry with a diamond ATR unit. HR-ESI-MS was performed with a Waters Q-TOF Micro system spectrometer (using H_3_PO_4_ for calibration and as internal standard). 1D and 2D NMR spectra were recorded at room temperature on the Bruker Avance II 400 MHz and Bruker Avance 500 MHz spectrometers, operating at 400 and 500 MHz for ^1^H and 100 and 125 MHz for ^13^C, respectively. The chemical shifts (δ) are reported in ppm relative to solvent signals δ_H_ 7.16 and δ_C_ 128.39 for C_6_D_6_, and δ_H_ 7.26 and δ_C_ 77.00 for CDCl_3_, while the coupling constants (*J*) are given in Hz. Vacuum liquid chromatography (VLC) separations were carried out on the Merck Silica gel 60G (Merck), while column chromatography (CC) was performed using the Silica gel 60 (230-400 mesh, Merck), silver nitrate-impregnated Silica gel 60 [30], and gel permeation on Sephadex LH-20 (GE Healthcare). TLC analyses were carried out using aluminium-backed silica gel 60 F_254_ (0,2 mm thickness, Merck). Chromatograms were visualized under a UV lamp at 254 nm then sprayed with vanillin and KMnO_4_/K_2_CO_3_/NaOH solution followed by heating. Preparative TLC (PTLC) was run on 20×20 cm glass-coated plates (1 mm thickness, Analtech) and doped TLC plates in MeCN:AgNO_3_ solution [31]. HPLC was performed on the Agilent 1260 Infinity Quaternary LC system, equipped with a Standard Autosampler (G1329B), Thermostated Column Compartment (G1316A TCC), a Diode Array Detector VL (G1315D**),** and a semi-preparative column (XTerra RP18, 10×150 mm, 5 μm i.d, Waters).

### Plant Material

The aerial parts of *Kaunia lasiophthalma* (Griseb) R.M. King and H. Robinson were collected on September 5th, 2009, near Independencia, Cochabamba, Bolivia, at coordinates 17º11.10′ S 66º43.58′ W and an elevation of 2943 m, during the flowering period. This aromatic herbaceous plant can grow up to 4 m in height and bears white-purple flowers. Lic. Modesto Zárate did the authentication, and voucher specimens have been deposited at Herbario Forestal Martín Cárdenas, Cochabamba (accession number MZ-3948).

### Extraction and Isolation

The air-dried and ground leaves (788.5 g) and flowers (1064.0 g) were extracted separately by maceration with 95% EtOH for 24 hours, two times at room temperature. After filtration, the combined extracts were concentrated under reduced pressure and the following crude extracts were obtained: 90.0 g from leaves and 91.0 g from flowers.

#### Leaves

The crude organic extract (90.0 g) was suspended in a mixture of H_2_O:MeOH (9:1, v/v, 500 ml) and partitioned between hexane (four times, 1:1, v/v), CH_2_Cl_2_ (two times, 1:1, v/v), and EtOAc (one time, 1:1, v/v). After evaporation of the solvent, the extracts weighed 22.68, 33.83, and 7.24 g, respectively. Subjection of the CH_2_Cl_2_ (18.7 g) to VLC (PE:EtOAc 1:0 to 0:1) gave four major fractions (A–D) based on TLC analyses. Purification of fraction B (10.8 g), using open CC (PE:CH_2_Cl_2_ 1:0 to 0:1; CH_2_Cl_2:_EtOAc 1:1) yielded nine fractions (B1–B9). Fraction B5 (579.6 mg) was chromatographed by flash CC (heptane:Me_2_CO 98:2 to 88:12) and combined into 13 fractions according to its TLC profile (B5.1–B5.13). Stigmasterol (52.4 mg) and **1** (35.6 mg) were obtained pure from B5.1 and B5.3, respectively. Further purification of B5.6 (80.0 mg) by CC (heptane:Et_2_O 100:0 to 95:5) and Sephadex LH-20 (MeOH) afforded **10** (6.3 mg) and **8** (32.9 mg). Fraction B7 (2.64 g) was applied on the Sephadex LH-20 CC (MeOH) to give six fractions (B7.1–B7.6). B.7.4 (631.0 mg) and B8 (948.6 mg) were submitted to chromatography on the Sephadex LH-20 CC (MeOH) to give **16** (46.1 mg) after recrystallization in MeOH. Fraction B7.5 (316.0 mg) and B7.7 (153.7 mg) were sequentially purified by the Sephadex LH-20 (MeOH), CC (PE:EtOAc 1:0 to 8:2), and PTLC (PE:EtOAc 6:4) giving **3** (1.7 mg) and **7** (18.9 mg), respectively. Fraction B9 (956.8 mg) was fractionated by CC (heptane:Me_2_CO 9:1) affording nine fractions (B9.1–B9.9). Each individual fraction B9.3 (139.4 mg) and B9.4 (470.0 mg) were further purified by the Sephadex LH-20 CC (MeOH) followed by CC (PE:EtOAc 1:0 to 8:2) to obtain **6** (10.0 mg) and **9** (80.0 mg). Compound **17** (59.0 mg) was recrystallized in CH_2_Cl_2_:MeOH from B9.6 (120.0 mg). Fraction C (589.0 mg) was separated by the Sephadex LH-20 CC (MeOH) to yield seven fractions (C1–C7). A mixture 1:1 of compounds **18** and **19** (26.0 mg) were obtained from C7 (330.0 mg).

#### Flowers

The flower extract (75.8 g) was partitioned as described above to yield the corresponding hexane (30.19 g), CH_2_Cl_2_ (26.52 g), and EtOAc (2.13 g) fractions, respectively. The CH_2_Cl_2_ fraction (24.0 g) was subjected to VLC (PE:EtOAc 1:0 to 0:1) and combined according to its TLC profile into nine fractions (A–H). Fraction B (1.23 g), containing mostly **1,** was dissolved in heptane:MeOH, filtered, concentrated, and recrystallized from heptane:Me_2_CO to afford pure **1** (318.0 mg). Fraction C (633.0 mg) was subjected to VLC (PE:EtOAc 1:0 to 0:1) to yield 11 fractions (C1–C11). C3 (360.0 mg) was dissolved in PE and centrifuged to obtain **10** (8.0 mg) as a white powder. The liquid residue was chromatographed by CC (PE:Et_2_O 1:0 to 2:3) giving eight fractions (C3.1–C3.8). Compound **8** (4.5 mg) was obtained from C3.5 (29.0 mg) by recrystallization from hexane:CH_2_Cl_2_ and **13** (2.7 mg) from C3.8 (17.8 mg) by HPLC (MeOH:H_2_O 3:7, t_R_=13.5 min, 240 nm, 1 ml/min). Subjection of fraction D (1.78 g) to VLC (PE:Et_2_O 1:0 to 0:1) afforded six fractions (D1–D6). Compound **12** (173.0 mg) was purified from D4 (390.0 mg) and D5 (386.0 mg) by recrystallization from heptane:Me_2_CO. The liquid residue (232.0 mg) was subjected to flash CC (heptane:CH_2_Cl_2_:Me_2_CO 10:10:1) to produce **7** (23.3 mg). D6 (470.0 mg) was applied to the Sephadex LH-20 CC (MeOH) to give **14** (36.0 mg). E (270.0 mg) was fractionated by VLC (heptane:Me_2_CO 1:0 to 5:1) to give seven fractions (E1–E7). E3 (151.0 mg) and E4 (642.0 mg) were combined and separated by flash CC (Silica gel doped with 10% AgNO_3_ w/w, PE:Et_2_O:Me_2_CO 5:5:1) to yield **9** (96.0 mg) and **6** (32.0 mg) as white needles. Sequential purification of E5 (1.02 g) was achieved by the Sephadex LH-20 (CHCl_3_:MeOH 1:1) and CC (heptane:CH_2_Cl_2_:Me_2_CO 2:17:1) to yield **15** (1.1 mg), **14** (16.0 mg), **9** (37.0 mg), and **7** (10.0 mg). Fraction F (1.53 g) was applied to VLC (hexane:Et_2_O 20:1, 1:3; hexane:Me_2_CO 99:1, 80:20) yielding 12 fractions (F1–F12). The CH_2_Cl_2_ soluble part of F4 (194.0 mg) was purified by VLC (CH_2_Cl_2_:Me_2_CO 1:0 to 4:1) giving three fractions (F4.1–F4.3). Each fraction was further purified to afford **11** (10.0 mg), **2** (2.9 mg), and **16** (4.7 mg), respectively. Repeated purification of G (950.0 mg) by VLC (CH_2_Cl_2_:Me_2_CO 98:2 to 0:1) and flash CC (CH_2_Cl_2_:Me_2_CO 9:1) afforded **4** (10.1 mg) by recrystallization from heptane:Me_2_CO. Fraction I (4.9 mg) was subjected to CC (CH_2_Cl_2_:Me_2_CO 6:4 to 0:1) yielding seven fractions (I1–I7). Sequential purification of I3 (1.06 g) by the Sephadex LH-20 (CHCl_3_:MeOH 1:1) and CC (CH_2_Cl_2_:Me_2_CO 6:4 to 0:1) produced **5** (9.7 mg) and **20** (2.5 mg).

(+)-(3aS*,5aR*,6S*,9S*,9aS*,9bS*)-9-Hydroxy-5a,9-dimethyl-3-methylidene-2-oxododeca-hydronaphtho[1,2-*b*]furan-6-yl acetate [(+)-*rel*-(1α,6α)-1-(Acetyloxy)-4-hydroxy-6,12-epoxyeudesm-11(13)-en-12-one, **3**]

Colourless gum; [α]d^25^ +70.8 (*c* 0.4, CHCl_3_); IR *v*_max_(cm^−1^) 3473, 2933, 2865, 2359, 2342, 1764, 1730, 1375, 1246, 1197, 1134, 1018, 966; ^1^H (500 MHz, C_6_D_6_), and ^13^C (125 MHz, C_6_D_6_) see Table 1; HR–ESI-MS m/z 309.1722 [M + H]^+^ (calcd. for C_17_H_25_O_5_ 309.1702).

(3aS*,5aR*,6S*,9aS*,9bS*)-5a-Methyl-3,9-dimethylidene-2-oxododecahydro-naphtho[1,2-b]furan-6-yl acetate [(+)-*rel*-(1α,6α)-1-(Acetyloxy)-6,12-epoxyeudesma-4(14),11(13)-dien-12-one, **8**]

Colourless oil; [α]d^25^ +170.4 (*c* 0.3, CHCl_3_); IR *v*_max_(cm^−1^) 2942, 1769, 1726, 1372, 1240, 1174, 1121, 1039, 1016, 969. ^1^H (500 MHz, CDCl_3_), and ^13^C (125 MHz, CDCl_3_) see Table 1; HR–ESI-MS *m/z* 291.1613 [M + H]^+^ (calcd. for C_17_H_22_O_4_ 291.1596).

### Preparation of Compound 21

10.0 mg (0.04 mmol) of **9** and 8.3 mg (0.048 mmol) methyl-L-cysteine ester hydrochloride were dissolved in methanol (1 mL) and the mixture was heated at 60°C and stirred. After 48 h, 1.6 mg (0.0093 mmol) methyl-L-cysteine ester was added and the reaction mixture was left for another 24 h. Then, the solvent was evaporated under reduce pressure and the dry residue dissolved in H_2_O (3 mL), and washed with CHCl_3_ (3 × 1.5 mL). The organic phase dried in vacuo yielded 15.5 mg of a yellow residue, which was purified by the Sephadex LH-20 (CHCl_3_:MeOH 1:1) to give 8.0 mg (0.021 mmol) of **21** as yellow oil. ^1^H NMR (400 MHz, CD_3_OD): δ 5.35 (m, 1H), 4.12 (t, *J* = 11.0 Hz, 1H), 3.75 (s, 3H), 3.69 (m, 1H), 3.59 (m, 1H), 3.00 (m, 1H), 2.94 (m, 1H), 2.88 (m, 1H), 2.84 (m, 1H), 2.76 (m, 1H), 2.28 (m, 1H), 1.98 (m, 1H), 1.96 (m, 1H), 1.95 (m, 2H), 1.76 (s, 3H), 1.66 (m, 2H), 1.26 (m, 1H), 0.89 (s, 3H). ^13^C (100 MHz, CD_3_OD): δ 179.5 (C-12), 175.4 (C-3′), 134.7 (C-4), 122.7 (C-3), 82.8 (C-6), 75.9 (C-1), 55.0 (C-2′), 52.7 (C-4′), 52.2 (C-5), 51.8 (C-11), 47.0 (C-7), 41.9 (C-10), 38.6 (C-1′), 35.9 (C-9), 33.5 (C-2), 31.6 (C-13), 24.1 (C-8), 23.6 (C-15), 11.3 (C-14). HR-ESI-MS *m/z* 406.1703 [M + Na]+ (calcd. for C_19_H_29_NO_5_SNa 406.1664).

### Biological Assay

#### Cell Culture

The L56Br-C1 cell line was established at the Department of Oncology, Clinical Sciences, Lund University, Sweden [32]. The JIMT-1 cell line was purchased from the German Collection of Microorganisms and Cell Cultures (Braunschweig, Germany) and the MCF-7, SK-BR-3, HCC1937, and MCF-10A cells were obtained from the American Tissue Type Culture Collection (Manassas, VA, USA). The cell lines were cultured as monolayers at 37°C in a humidified incubator with 5% CO_2_ in air. The MCF-10A cells have a population doubling time of 15 hours, the JIMT-1 cells 24 hours, and the other four cell lines around 35 hours.

The L56Br-C1, MCF-7, SK-BR-3, and HCC1937 cells were cultured as described by Holst et al [33]. JIMT-1 cells were cultured in DMEM/Ham’s F12 medium supplemented with 10% fetal calf serum, non-essential amino acids (1 mM), insulin (10 μg/ml), penicillin (100 U/ml), and streptomycin (100 μg/ml). MCF-10A cells were maintained in RPMI 1640 medium supplemented with 10% heat-inactivated fetal calf serum, non-essential amino acids (1 mM), insulin (10 μg/ml), epidermal growth factor (20 ng/ml), cholera toxin (50 ng/ml), hydrocortisone (250 ng/ml), penicillin (100 U/ml), and streptomycin (100 μg/ml). The MCF-10A and JIMT-1 cell lines were sub-cultured twice a week, while the L56Br-C1, MCF-7, SK-BR-3, and HCC1937 cells were sub-cultured once a week with an additional change of growth medium once a week.

#### Dose-Response Assay

Stock solutions (10 or 100 μM) of the compounds were made in 100% DMSO. These were further diluted in PBS to obtain the correct concentrations used for the MTT assay. Appropriate DMSO controls were used. In general, the highest DMSO concentration was 0.1%, however, when treating with 100 μM and starting from a 10 μM stock in 100% DMSO, a final concentration of 1% DMSO was used as the control.

The MTT assay was performed as previously described [34]. Briefly, the cells were trypsinized and counted in a hemocytometer. Aliquots of 180 μl cell suspensions containing 3000 (MCF-10A) and 6000 (MCF-7, SK-BR-3, JIMT-1, L56Br-C1, and HCC1937) cells were seeded in the wells of 96-well plates. Compounds were added 24 hours after seeding to allow the attachment of cells. A concentration range between 0.1 to 100 μM was used in the MTT assays and appropriate DMSO controls. At 72 h of drug treatment, 20 μl of MTT solution (5 mg/ml MTT in PBS) was added to each well and the 96-well plates were returned to the CO_2_ incubator for 1 hour. The MTT-containing medium was removed. The blue formazan product formed by the reduction in live attached cells was dissolved by adding 100 μl of 100% DMSO per well after removal of the MTT-containing medium. The plates were swirled gently at room temperature for 10 minutes to dissolve the precipitate. Absorbance was monitored at 540 nm in a Labsystems iEMS Reader MF (Labsystems Oy, Helsinki, Finland) using the DeltaSoft II v.4.14 software (Biometallics Inc., Princeton, NJ, USA). Dose-response curves were drawn based on the % of the control in Excel. The IC_50_ was deduced from the curves.

## Supporting Information



## Figures and Tables

**Tab. 1:** ^1^H and ^13^C NMR data of compounds **3** (C_6_D_6_) and **8** (CDCl_3_)

Position	3		8	
	
	^1^H (*J*, Hz)	^13^C	^1^H (*J*, Hz)	^13^C
1	4.67 br dd (2, 2)	77.2	4.68 br dd (2, 3)	76.4
2β	1.99 dddd (13, 13, 4.5, 2.2)	23.0	1.86 dddd (13.9, 13.4, 5.1, 2.7)	27.1
2α	1.53 m		1.81 m	
3β	1.15 m	36.2*	2.20 ddd (13.5, 5.1, 1.9)	30.5
3α	1.53 m		2.32 ddd (13.5, 13.4, 5.4)	
4	–	71.0	–	143.2
5	1.57 d (11.2)	50.5	2.79 d (11.0)	48.7
6	3.70 dd (11, 11)	79.8	4.00 dd (11, 11)	79.8
7	1.76 m	50.7	2.55 ddddd (12, 11, 3, 3, 3.4)	49.3
8β	1.26 m	21.4	1.61 dddd (12, 12, 11, 3.4)	21.2
8α	1.03 m		2.08 m	
9β	0.84 m	36.2*	1.42 ddd (13.0, 2.8, 3.2)	33.1
9α	1.24 br ddd (14, 14, 4)		1.72 ddd (13.0, 13.0, 4.0)	
10	–	40.7	–	41.7
11	–	140.4	–	139.1
12	–	170.0	–	170.5
13	5.94 d (3.2)	115.9	6.09 d (3.2)	117.0
	4.79 d (3.2)		5.40 d (3.2)	
14	0.92 s	20.0	0.92 s	18.0
15	1.29 s	32.9	5.00 br s	110.2
			4.87 br s	
1′	–	169.7	–	170.4
2′	1.74 s	21.1	2.12 s	21.2

δ (ppm) 500 MHz for ^1^H and 125 MHz for ^13^C; multiplicities; *J* values (Hz) in parentheses;

* May have interchanged.

**Tab. 2:** Cytotoxicity (IC_50_ in μM) of compounds **1**, **3**, **6–9**, **11**, **12**, **14**, **16**, and **21**

Comp.	HCC1937	JIMT-1	L56Br-C1	MCF-7	SK-BR-3	MCF-10A
	μM	μM	μM	μM	μM	μM
1	2.2/4.2^b^	6.2/6.3^b^	3.7/4.8^b^	5.3±1.4^c^	2.0±1.0^c^	20.0^a^
3	23.0^a^	18.0^a^	9.3^a^	27.0^a^	10.1^a^	38.0^a^
6	10.0^a^	8.5/12.0^b^	11.0^a^	16.4±7.1^c^	5.2±1.6^c^	17.0^a^
7	12.0/18.5^b^	12.0/14.0^b^	9.0/10.1^b^	23.0/30.0^b^	7.6/6.4^b^	22.0^a^
8	3.2/7.5^b^	6.3/7.0^b^	8.4/12.0^b^	11.0±1.7^c^	4.7±1.1^c^	24.0^a^
9	8.1±1.8^c^	6.9/7.2^b^	12.3±5.5^c^	9.7±0.9^c^	3.1±0.6^c^	17.0^a^
11	4.8/7.8^b^	10.0/13.0^b^	3.6^a^	10.1/18.0^b^	4.2±2.1^c^	24.0/21.0^b^
12	6.0/7.8^b^	7.1/10.1^b^	6.0/10.1^b^	7.1/8.3^b^	4.2±1.1^c^	17.0/38.0^b^
14	3.0/5.2^b^	7.5/8.1^b^	5.8/10.0^b^	3.3/4.3	2.5±0.4^c^	20.0/23.0^b^
16	24.0^a^	13.0/23.0^b^	16.0^a^	27.4±17.2^c^	9.9±3.0^c^	29.0^a^
21	100.0^a^	>100	73.0^a^	>100	51.0/40.0^b^	41.0^a^

Values from:

a one dose-response curve,

b two dose-response curves,

c three or more dose-response curves.
