# Supplementary material for: Cytotoxic Sesquiterpene Lactones from Kauna lasiophthalma Griseb
Source: Sci Pharm. 2014 Jan 18;82(1):147–60. doi: 10.3797/scipharm.1310-18 (PMC3951225; doi:10.3797/scipharm.1310-18)

Scientia Pharmaceutica www.scipharm.at

Open Access

Supporting Information to

Cytotoxic Sesquiterpene Lactones from *Kauna lasiophthalma* Griseb

Eliana M. MALDONADO, Daniel SVENSSON, Stina M. OREDSSON, Olov STERNER

Published in Sci Pharm. 2014; 82: 147–160 doi:10.3797/scipharm.1310-18

Available from: http://dx.doi.org/10.3797/scipharm.1310-18

© Maldonado *et al.*; licensee Österreichische Apotheker-Verlagsgesellschaft m. b. H., Vienna, Austria.

This is an Open Access article distributed under the terms of the Creative Commons Attribution License (http://creativecommons.org/licenses/by/3.0/), which permits unrestricted use, distribution, and reproduction in any medium, provided the original work is properly cited.

Table of Contents

^{1}H-NMR of Compound **3**
^{13}C-NMR of Compound **3**
^{1}H-NMR of Compound **8**
^{13}C-NMR of Compound **8**

^{1}H-NMR of Compound 3

4-*epi*-1α-acetoxy arbusculin A (3)

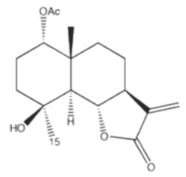

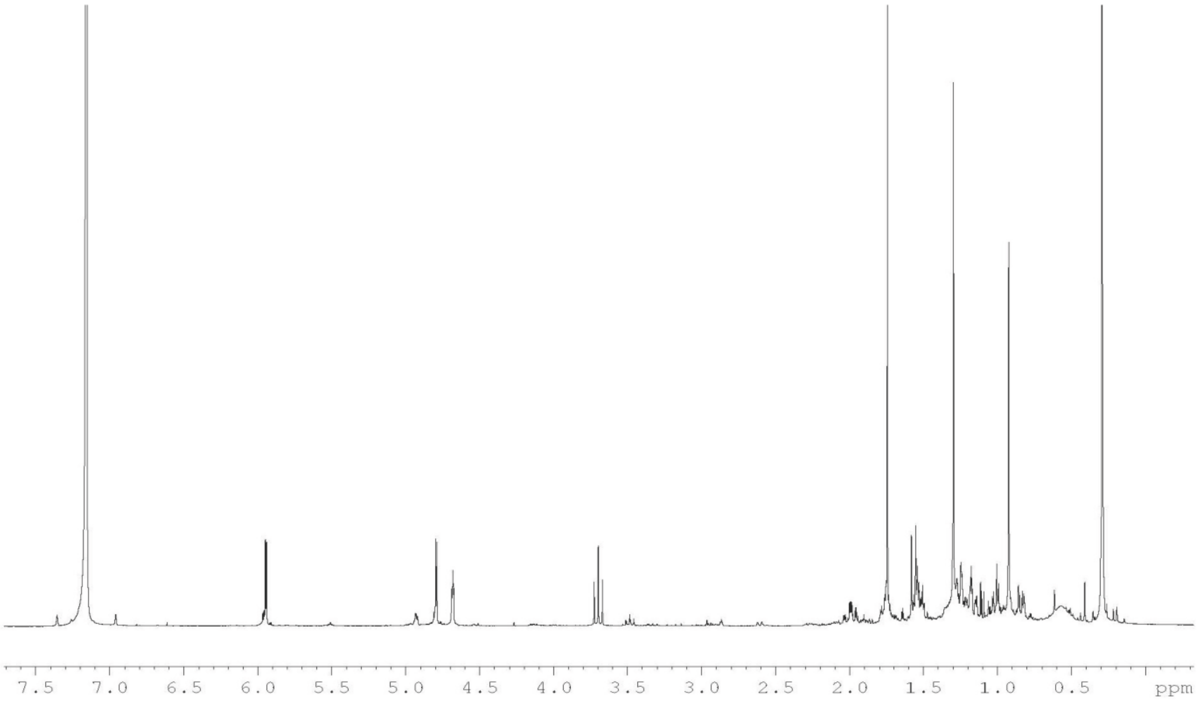

^{13}C-NMR of Compound 3

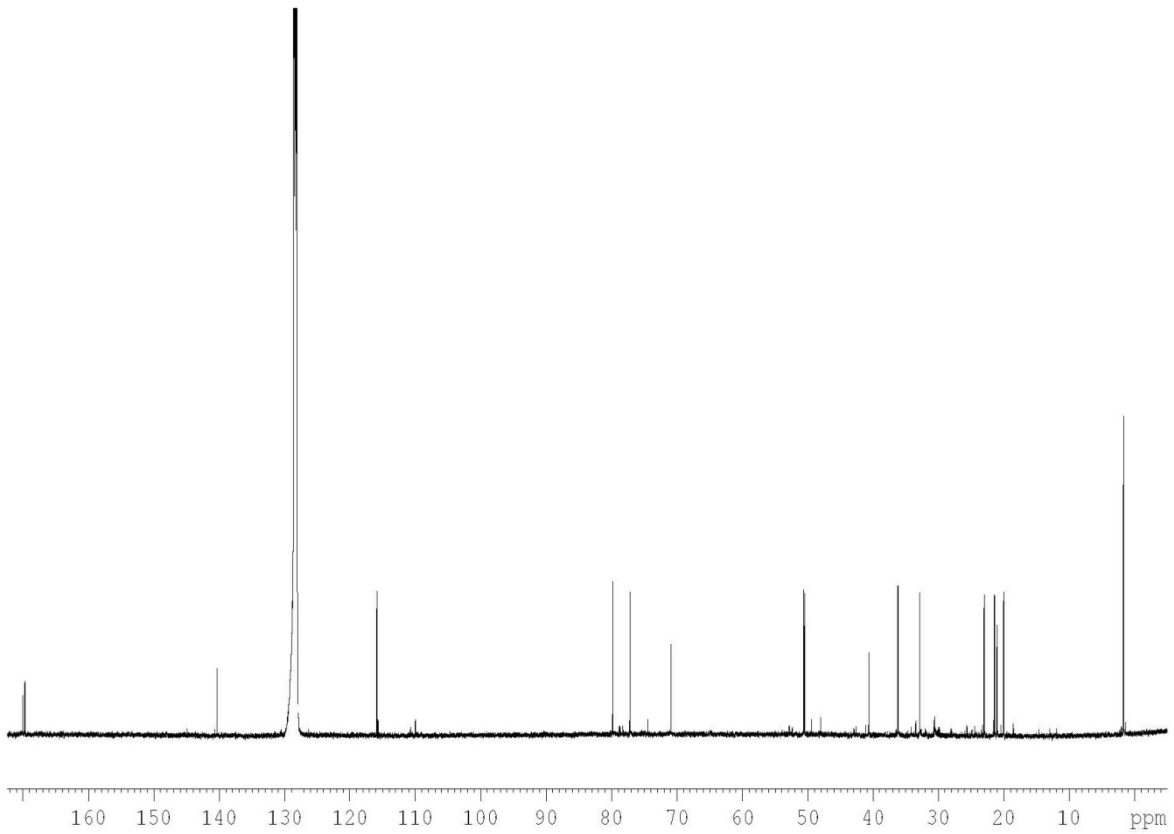

^{1}H-NMR of Compound 8

acetyl 1-*epi*-reynosin

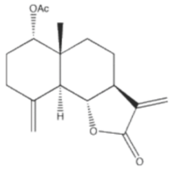

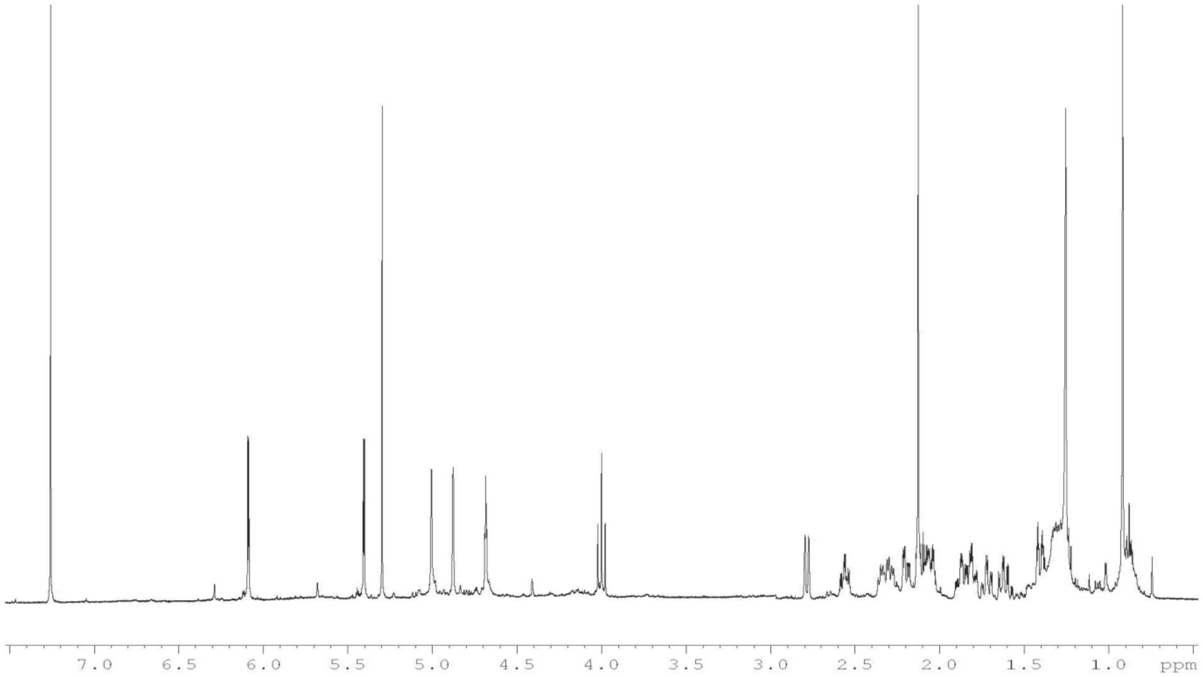

^{13}C-NMR of Compound 8

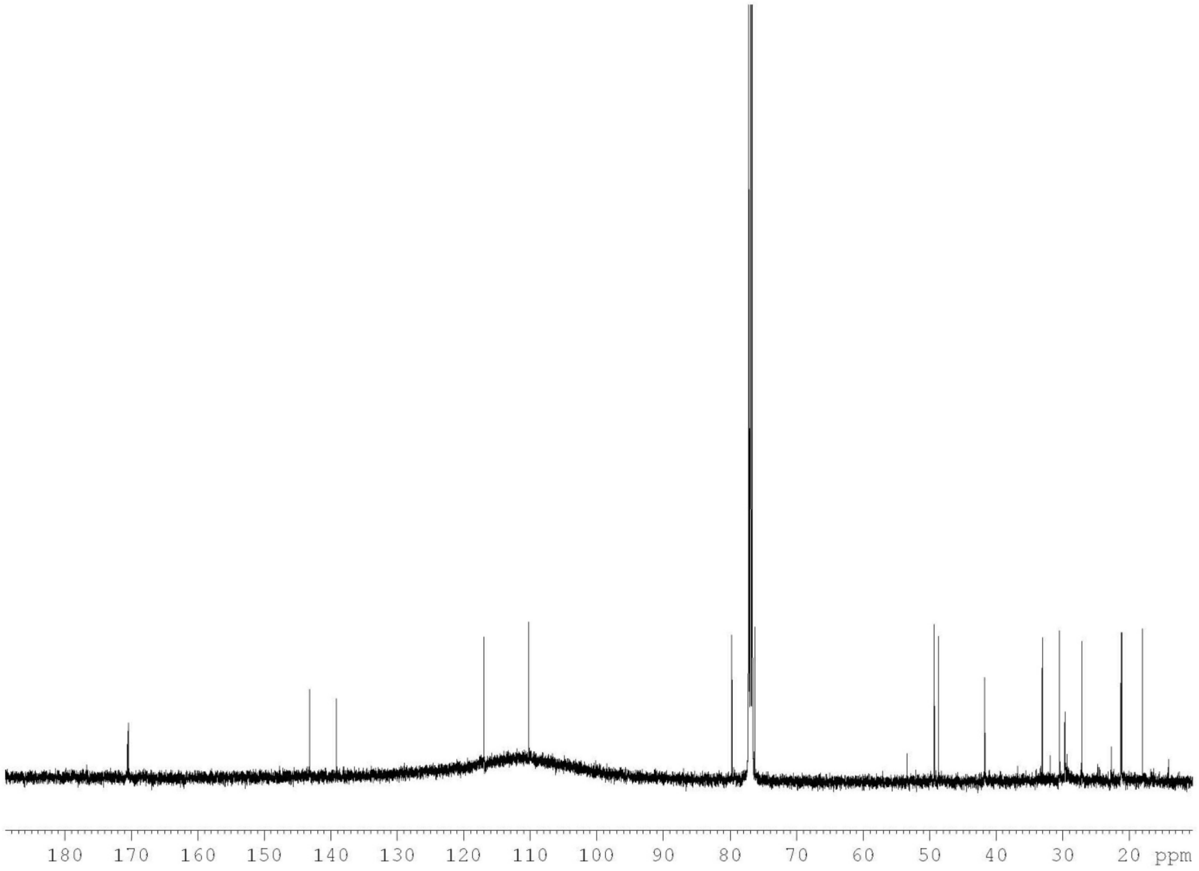

Supplement: Supplementary file 1 [file Scipharm.2014.82.147_supporting_information.pdf]
